# In-silico identification of bacterial key-genes directly or indirectly associated with the development and progression of colorectal cancer for exploring anti-bacterial agents

**DOI:** 10.1371/journal.pone.0343565

**Published:** 2026-06-26

**Authors:** Nibas Kumar Pal, Md. Kaderi Kibria, Tasfia Noor, Md. Feroj Ahmed, Md. Shariful Islam, Md. Foysal Ahmed, Md. Abdul Latif, Mohammad Ali, Md. Al Noman, Dipto Kundu, Ovi Sharma, Md. Nurul Haque Mollah

**Affiliations:** 1 Bioinformatics Lab (Dry), Department of Statistics, University of Rajshahi, Rajshahi, Bangladesh; 2 Department of Computer Science and Engineering, Rajshahi University of Engineering and Technology, Rajshahi, Bangladesh; 3 Department of Statistics and Data Science, Barishal University, Barishal, Bangladesh; 4 TMSS Medical College, Bogura, Bangladesh; Universita degli Studi della Campania Luigi Vanvitelli, ITALY

## Abstract

Colorectal cancer (CRC), which includes malignancies of the colon and rectum, constitutes a major global health challenge. Though there are several drugs that targets CRC-related genes/proteins, but their performance is not yet reach to the satisfactory level. Moreover, their effectiveness gradually decreases over time with long-term use, a phenomenon known as drug resistance. Therefore, it is required to explore new alternative candidate drugs against CRC. Several studies recommended CRC-related dysregulated host-genes guided candidate drugs. However, microbiome guided drug discovery particularly targeting bacterial key genes (bKGs) within CRC-associated gut microbiota remains very limited. This study aims to identify bKGs as antibacterial targets within CRC-associated bacterial taxa for exploring anti-bacterial agents. At first, we analysed a 16S rRNA-seq profile dataset that contained 24 CRC and 50 healthy samples, where beta diversity analysis results showed significant differences in bacterial compositions between CRC and HC groups. Differential abundance analysis with threshold values at |log_2_FC| > 1.0 and adjusted *p*-value < 0.05 identified 42 significantly altered bacterial taxa of which *Bacteroides fragilis, Bacteroides ovatus, Bacteroides uniformis,* and *Flavonifractor plautii* were prioritized based on effect size and published literature reporting their association with CRC. Further, an integrative subtractive genomics and protein-protein interaction (PPI) network analyses was used to identify top-ranked 10 essential bKGs (*ribD, ribBA, murA, alr, hisI, hisE, hisD, hisG, hisH,* and *hisB*) from these four CRC-associated bacterial taxa as putative antibacterial targets. Finally, three candidate drug molecules (Sulfasalazine, Aminoglutethimide, and Tipiracil) were recommended as the preliminary bKGs-guided candidate anti-bacterial agents for CRC through molecular docking and ADME/T analyses. Further experimental and clinical validation is required to establish these compounds as the effective drugs targeting the bKGs for CRC. Thus, these findings may provide insights for developing innovative anti-bacterial treatment approach relevant to CRC.

## 1. Introduction

**Fig 1:**
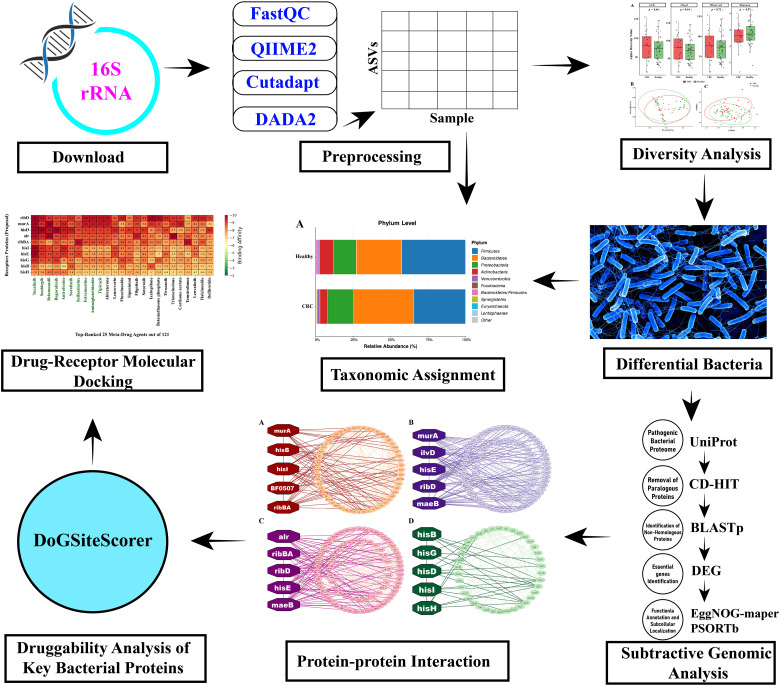
Workflow of the study.

Colorectal cancer (CRC), defined as malignancy originating in the colon or rectum, is a major global health concern and remains one of the leading causes of cancer-related mortality worldwide [1,2]. Despite substantial advances in early detection and therapeutic interventions, the global burden of CRC continues to rise, particularly in low and middle-income countries [3]. In 2020, CRC accounted for more than 1.9 million new cases and approximately 930,000 deaths, ranking as the third most commonly diagnosed cancer and the second deadliest malignancy globally [4,5]. Its global incidence is projected to reach 3.2 million new cases by 2040 [3]. Current treatment strategies primarily focus on host-driven molecular pathways [6]. Many patients experience limited therapeutic response, disease recurrence or treatment failure over time due to drug resistance, tumor heterogeneity and adverse side effects [7,8]. Although cumulative genetic and epigenetic alterations may contribute to CRC development, host-derived mechanisms alone do not fully explain CRC initiation and progression. Recent studies increasingly highlight the gut microbiome as a potential factor associated with colorectal carcinogenesis [9]. Under physiological conditions, the intestinal microbiota plays an essential role in maintaining host metabolic balance, immune regulation and epithelial integrity [10]. Disruption of this balanced microbial ecosystem referred to as dysbiosis has been associated with the expansion of potentially harmful bacterial taxa and altered host-microbe interactions, which have been implicated in CRC development though the causal and directional nature of these relationships remains to be fully established [11,12]. High-throughput sequencing technologies, including 16S rRNA gene sequencing and shotgun metagenomics, have significantly enhanced our understanding of CRC-associated microbial communities [13]. Evidence suggests bidirectional association between the host genome and gut metagenome, where changes in microbial composition have been linked to alterations in host molecular pathways and, conversely, host genetic variation has been associated with differences in microbial ecology [14,15]. Pathogenic bacteria may further play a role in disease progression through the expression of virulence proteins that potentially interact with host proteins, subvert immune responses, disrupt cellular signaling, and promote bacterial survival and replication, though whether these interactions directly contribute to disease progression remains to be functionally established [16–18]. Several 16S rRNA-based studies have identified distinct bacterial taxa associated with CRC, supporting the investigation of their functional gene content as putative targets for microbiome-guided therapeutic strategies [19–23]. Despite these advances in microbial profiling, the functional genetic determinants particularly essential genes unique within CRC-associated bacterial taxa, designated herein as bacterial key genes (bKGs), and their functionally encoded protein products designated as bacterial key proteins (bKPs), remain poorly characterized. While host genome-guided therapeutic strategies for CRC have been extensively explored [24,25], microbiome-guided drug discovery particularly targeting essential bKGs within CRC-associated bacteria remains largely unexplored. Considering the potential role of bacterial genes in mediating host-microbe interactions, identifying essential bKGs in CRC-associated bacteria as antibacterial targets represents a promising avenue for developing antibacterial therapeutic strategies against CRC. To address this gap, the present study employed a comprehensive in-silico framework including 16S rRNA-based microbial profiling, subtractive genomics, PPI network analysis, molecular docking, and ADME/T analysis to identify bKGs as putative antibacterial targets in CRC associated bacterial species, and to explore bKG-guided antibacterial drug candidates. By integrating 16S rRNA sequencing-based microbial profiling with functional genomics and computational drug screening, this study aims to bridge the gap between CRC-associated microbiome composition and microbiome-guided antibacterial drug discovery. The findings are expected to provide novel insights into putative antibacterial intervention strategies and contribute to the development of innovative therapeutic candidates for CRC targeting essential bKGs. The entire workflow of the study depicted in **Fig 1**.

## 2. Materials and methods

### 2.1. Data source and description

In this study, publicly available 16S rRNA gene sequencing data from human stools, along with their corresponding metadata were analyzed. Sequence reads were retrieved from the European Nucleotide Archive (ENA) under BioProject accession number PRJEB77293 (https://www.ebi.ac.uk/ena/browser/view/PRJEB77293). The original dataset comprised 148 samples with 36 colonic tissue specimens and 112 stool samples. Clinical metadata (e.g., age, BMI, diet, medication use, and antibiotic exposure) were not available for the publicly accessible dataset used in this study. For the present analysis, only stool-derived microbiome profiles from clearly defined CRC case and healthy control (HC) were included. Specifically, 24 stool samples from CRC patients and 50 samples from HC representing the Sri Lankan population were selected [26].

### 2.2. Raw 16S rRNA sequence data processing

Raw 16S rRNA sequencing reads were considered to proper quality control and preprocessing prior to downstream analysis. As the data were generated within a single study under a consistent experimental framework, batch effects were not considered. Initial quality assessment was performed using FastQC (v0.12.1) [27] to detect low-quality reads, adapter contamination, and overrepresented sequences. Subsequent processing was carried out using QIIME 2 (amplicon-2024.10) [28]. Primer and adapter sequences, along with low-quality bases, were removed using the Cutadapt plugin [29] integrated within the QIIME 2 framework, a commonly used framework for microbial community studies. This initial processing was essential for maintaining the precision and reliability of microbiome assessment in both CRC and HC. Afterward, the paired end reads underwent quality filtering, trimming and denoising through the DADA2 [30] plugin within the QIIME 2 environment, allowing only high-quality sequences to be retained for downstream analyses. The denoised paired-end reads were then merged through the DADA2 workflow, preserving the default overlap and mismatch allowance to ensure accurate sequence merging. After merging, chimera removal and dereplication were performed using the DADA2 plugin within QIIME 2 to infer exact amplicon sequence variants (ASVs). The resulting ASV table contains the abundance of each unique sequence feature across all samples. This table formed the basis for subsequent downstream analyses.

### 2.3. Microbial diversity analysis

The assessment of bacterial diversity was conducted utilizing R software (v4.4.2). To evaluate alpha diversity, the analysis incorporated Chao1, Observed species, ACE, and Shannon metrics [31–33] calculated through the “phyloseq (v1.50.0)” R package [34,35]. The Chao1, Observed, and ACE indices quantify bacterial richness [36,37], whereas the Shannon index captures both species richness and community evenness [36]. Diversity assessments were conducted on rarefied count data standardized to the sequencing depth equals the minimum read count, using the “phyloseq” R package. Statistical comparisons of alpha diversity across groups were performed using the non-parametric Wilcoxon rank-sum test [38]. Conversely, beta diversity, which reflects differences in microbial composition between samples, was evaluated using the Bray-Curtis dissimilarity metric [39] based on the rarefied ASV abundance table, computed through the “phyloseq” package in R. Principal Coordinate Analysis (PCoA) was employed to graphically represent variations in microbial community structure among samples [40]. Beta diversity was also assessed using Jaccard dissimilarity and visualized through Non-metric Multidimensional Scaling (NMDS) [41]. Differences in bacterial community composition between the CRC and HCs were assessed using PERMANOVA based on Bray-Curtis and Jaccard distance metrics, implemented in the vegan (v2.6-10) package in R with 999 permutations [42].

### 2.4. Taxonomic profiling

Probabilistic taxonomic classification of the ASVs were carried out using a Naive Bayes classifier trained in QIIME 2, based on the Greengenes-SILVA-RDP (GSR) database, a manually curated and optimized 16S rRNA gene reference database [43]. Reference sequences and corresponding taxonomy for the V4 region (primers 515F/806R) were utilized to train the model through the feature-classifier plugin in QIIME 2 [44]. Taxonomic classification of ASVs was then inferred using the classify-sklearn method, though V4 amplicon-based classification provides probabilistic taxonomic assignments rather than definitive species identification and resolution at the species level may be limited particularly for complex genera. Relative abundance profiling was performed across hierarchical taxonomic levels (phylum, class, order, family, genus, and species) using QIIME 2 [45].

### 2.5. Differential abundance testing

Differentially abundant bacterial taxa between CRC and HC group were identified using a Zero-Inflated Gaussian Mixture Model (ZIGMM) [46] through the “metagenomeSeq”(version: 1.48.1) R package [47]. Taxa with adjusted *p*-value < 0.05 and |log_2_FC| > 1 were defined as significantly differentially abundant. The false discovery rate (FDR) was controlled using the Benjamini-Hochberg correction to account for multiple hypothesis testing [48]. Taxa having greater effect size, species-level taxonomic annotations, and support from published articles about their associations with CRC, were considered to prioritize for further investigation.

### 2.6. Subtractive genomic approach for bKGs identification in CRC-associated bacteria

The full proteome of the selected bacterial taxa, along with the human reference proteome, were collected from UniProt database [49]. Reference proteomes were used as functional approximations, as the actual bacterial strains present in the patient samples remain uncharacterized and may differ from the reference strains analyzed. To remove paralogous sequences, the protein sequences were clustered using CD-HIT at a 60% sequence identity cutoff (word size = 4), ensuring that only non-paralogous proteins were retained for further analysis [50]. To exclude proteins with host similarity, all non-paralogous proteins were screened against the human proteome using BLASTp. Proteins sharing sequence identity greater than 30% and query coverage of at least 70% with any human proteins at an E-value threshold of 1 × 10 ⁻ ^5^ or lower were considered human homologs and excluded from further analysis [51]. Essential bacterial proteins were predicted by BLASTp searches against the Database of Essential Genes (DEG 10) [52], applying stringent thresholds of sequence identity ≥40%, query coverage ≥80%, bit score ≥100, and E-value ≤1 × 10 ⁻ ^10^ to select crucial proteins for bacterial survival [53–55]. Proteins failing to satisfy any of these conditions were excluded from downstream analysis. Functional characterization of the sequences covering enzyme commission (EC) numbers, KEGG pathway assignments was carried out with eggNOG-mapper [56]. PSORTb (v3.0) was employed to predict the subcellular locations of the candidate proteins, enabling distinction between cytoplasmic and membrane-associated proteins for effective drug-target prioritization [57–59]. Protein-protein interaction (PPI) networks of the cytoplasmic proteins of each taxa were generated using the STRING database with medium interaction confidence (0.4) and subsequently visualized in Cytoscape. [60,61]. Proteins exhibiting high interaction connectivity within the network were identified as hub proteins based on the Degree topological measure using the cytoHubba plugin in Cytoscape and considered potential therapeutic targets [62].

### 2.7. Druggability analysis of bKGs

Druggability of the target bKGs was analyzed using DoGSiteScorer [63], a pocket-detection and scoring module integrated into the ProteinPlus platform. The tool identifies potential ligand-binding cavities through a Difference-of-Gaussian-based segmentation procedure, which isolates regions of the protein surface likely to support small-molecule interaction. For each protein, DoGSiteScorer computes a set of geometric and physicochemical features, including pocket volume and surface area, drug score (0–1), and simple score which provide an overall estimate of the pocket’s suitability for binding drug-like compounds. Pockets with a drug score ≥ 0.5 were considered to have greater likelihood of being modulated by drug-like molecule [64]. When multiple pockets within a protein achieved comparable drug scores, the pocket with the greatest volume, surface area, and simple score was reported.

### 2.8. Molecular docking

To explore possible therapeutic agents for CRC, 121 drug compounds associated with CRC were intentionally extracted from the DrugBank database for drug repurposing perspective [65], a well-established and cost-effective strategy in computational drug discovery [66]. This is further supported by the evidence that non-antibiotic drugs can exhibit off-target antimicrobial activity [67], providing rationale for investigating CRC associated compounds as potential inhibitors of targets enriched in CRC-associated microbiota. The 3D structures of chosen drug molecules were obtained from the PubChem database [68], while structural models of the bacterial target proteins were sourced from AlphaFold [69], and SWISS-MODEL [70] databases. All molecular structures were systematically prepared prior to the molecular docking. The selected ligands were subjected to energy minimization, torsion tree generation, and charge assignment using Avogadro [71] and AutoDockTools4 [72]. Protein structures were refined by eliminating crystallographic water molecules, non-essential heteroatoms, and extraneous protein chains using Discovery Studio [73,74], followed by hydrogen addition, Kollman charge assignment, and grid box preparation in AutoDockTools4 [72]. Molecular docking of the preprocessed drug candidates with the target protein structures was performed using AutoDock Vina [75]. Blind docking was performed by defining a grid box encompassing the entire protein surface, allowing AutoDock Vina to globally explore all possible binding cavities without prior assumptions regarding active site location. The Binding Affinity Scores (BAS) from each docking result were assessed to identify the compounds showing the strongest predicted interactions with the target proteins.

### 2.9. Drug-likeness and ADME/T analysis

To evaluate the pharmacokinetic and toxicity profiles of the top ten candidate compounds, drug-likeness and ADME/T analyses were performed using the Deep-PK [76] and pkCSM [77]. Compound structures were submitted in Simplified Molecular Input Line Entry Specification (SMILES) format as input. Drug-likeness was assessed based on Lipinski’s Rule of Five [78], which defines acceptable physicochemical boundaries as molecular weight ≤ 500 Da, hydrogen bond donors ≤ 5, hydrogen bond acceptors ≤ 10, and lipophilicity (logP) ≤ 5. ADME/T properties including absorption, distribution, metabolism, excretion, and toxicity parameters were predicted and compounds satisfying both drug-likeness and ADME/T criteria were shortlisted as potential candidates for further consideration. The parameters of gut-targeted antibacterial activity including intestinal luminal stability, bacterial membrane uptake, luminal availability, and activity under anaerobic conditions were not assessed in this study. Therefore, ADME/T findings are preliminary filters for drug-likeness and safety assessment, rather than definitive indicators of suitability for gut-targeted antibacterial activity.

### 2.10. Validation of docking protocol using decoy molecules

To assess docking specificity, 40 decoy molecules were generated for each proposed drug compound as negative controls using the Directory of Useful Decoys Enhanced (DUDE-Z) [79], which provides high-quality property-matched decoys using the ZINC database (S15 Table). Molecular docking was subsequently performed between each target protein and its corresponding decoy ligands. Docking reliability was evaluated by comparing binding affinity of the proposed drug compounds against their corresponding decoy sets, confirming that active compounds consistently achieved superior binding affinities relative to decoy molecules across all target proteins [80].

### 2.11. Binding site assessment of final selected complexes

To assess binding site consistency of the final shortlisted compounds, a post-docking evaluation was performed for the ADME/T-screened ligands in complex with the two principal target proteins. Potential druggable regions (drug score >0.5) within the protein structures were identified using DoGSiteScorer. Subsequently, the spatial agreement between docked poses and predicted cavities was analyzed to evaluate binding site correspondence.

### 2.12. Molecular dynamics (MD) simulation

To examine the dynamic behavior of the most promising protein-ligand complexes, molecular dynamics (MD) simulations were performed using YASARA software [81]. The three ligands Sulfasalazine, Tipiracil, and Aminoglutethimide which demonstrated superior docking scores, satisfied Lipinski Rule of Five criteria, and exhibited favorable ADME/T profiles, were selected for 100 ns simulations of their respective protein-ligand complexes under physiological environments. Trajectory data were recorded at 100 ps intervals and subsequently analyzed using YASARA macros [82], alongside the SciDAVis software package (http://scidavis.sourceforge.net/). To further evaluate the stability of the interactions throughout the simulation, binding free energies (ΔGbind) were determined at 100 ps intervals employing the MM-PBSA approach implemented within YASARA [83].

## 3. Results

### 3.1. Preprocessing of bacterial 16S rRNA-sequence data

A total of 74 stool samples (24 CRC patients and 50 healthy controls) were included in the analysis. Following sequence processing, a high proportion of paired end reads (81%) were successfully merged, resulting in a feature table comprising 1,291 amplicon sequence variants (ASVs) across all samples. The dataset contained a total of 1,308,541 reads, with a median feature frequency of 17,981 counts per sample. Read counts ranged from 7,101 to 25,675 per sample, indicating adequate sequencing depth across samples. Prior to downstream analyses, unclassified and unassigned ASVs at the phylum level including ‘Unclassified Bacteria’ and ‘Unclassified Archaea’ were removed from the feature table using the “dplyr” (v1.1.4) package in R. After removing these, the feature table comprised 1,276 ASVs across 74 samples with median feature frequency of 17,980 counts per sample. The highest read count in a sample was 25,527 while the lowest was 7,101. Rarefaction analysis showed a progressive increase in ASV richness with greater sequencing depth, and the eventual plateau of the curves indicated that sampling effort was adequate and captured the majority of microbial diversity. Accordingly for diversity analysis, all samples were rarefied to the minimum sample read count. This ensured an even sampling effort for alpha and beta diversity comparisons. For differential abundance analysis, ASVs with prevalence below 5% of samples were excluded to minimize noise and retain biologically meaningful features. The final filtered feature table was normalized to provide a robust foundation for identifying taxa significantly associated with CRC.

### 3.2. Bacterial diversity analysis

**Fig 2:**
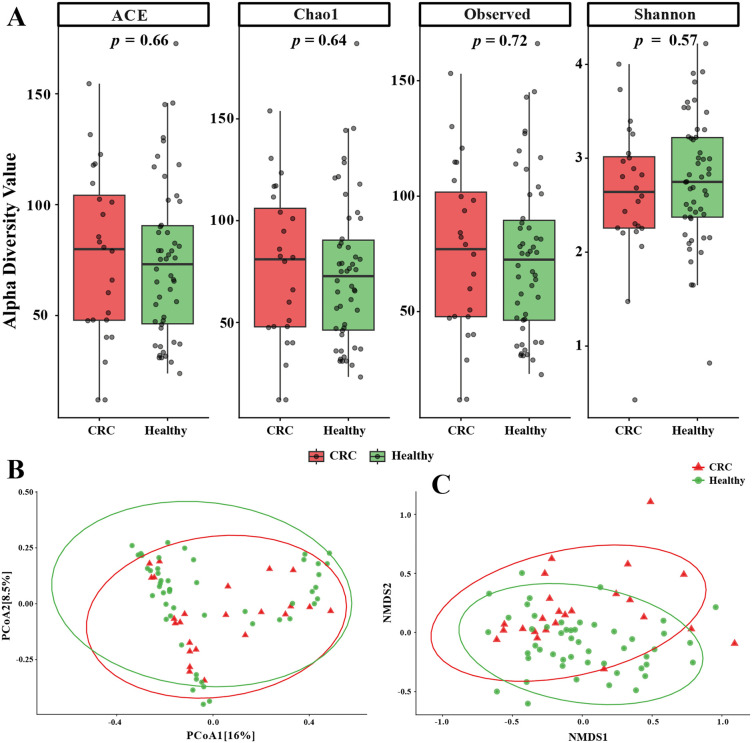
Alpha and beta diversity of the microbiota structure in CRC patients and HC. (A) Boxplots indicate no significant difference in alpha diversity indices (ACE, Chao1, Observed species, Shannon between CRC and HC. (B) and (C) highlight the Beta diversity using Bray-Curtis and Jaccard dissimilarity exhibiting significantly distinct microbial community composition.

Alpha and beta diversity analyses were performed to compare bacterial composition between HC and CRC samples. Alpha diversity was assessed using the Chao1, Observed, ACE, and Shannon indices (**Fig 2A**), and group differences were evaluated using the Wilcoxon rank-sum test. No statistically significant differences in alpha diversity were observed between CRC and HC across any of the indices assessed (Observed: *p-*value = 0.72; Shannon: *p*-value = 0.57; Chao1: *p*-value = 0.64; ACE: *p*-value = 0.66), suggesting that overall bacterial richness and evenness were comparable between groups.

Consequently, beta diversity was assessed using Bray-Curtis distance and Jaccard dissimilarity; the results were visualized using PCoA plots and NMDS plot respectively (Fig 2B-2C). Both metrics revealed different community composition patterns between groups. PERMANOVA test based on both metrics indicated significant differences in bacterial community composition between CRC and HC groups (Bray-curtis: F = 4.076, *p*-value = 0.0472, Jaccard-dissimilarity: F = 1.622, *p*-value = 0.007). Though the PERMANOVA result based on Bray-Curtis dissimilarity showed borderline significance (*p*-value = 0.0472), community-level differences were additionally supported by Jaccard dissimilarity analysis (*p*-value = 0.007), providing complementary evidence for microbiome compositional separation between groups. A test for homogeneity of group dispersions based on Bray-Curtis distance (Bray-curtis: F = 0.00998, *p*-value = 0.92) and Jaccard dissimilarity (Jaccard: F = 0.207, *p*-value = 0.65) indicated no significant difference in variance between groups, suggesting that the observed differences were not attributable to within-group variability, supporting the reliability of the PERMANOVA results.

### 3.3. Taxonomic profiling

**Fig 3:**
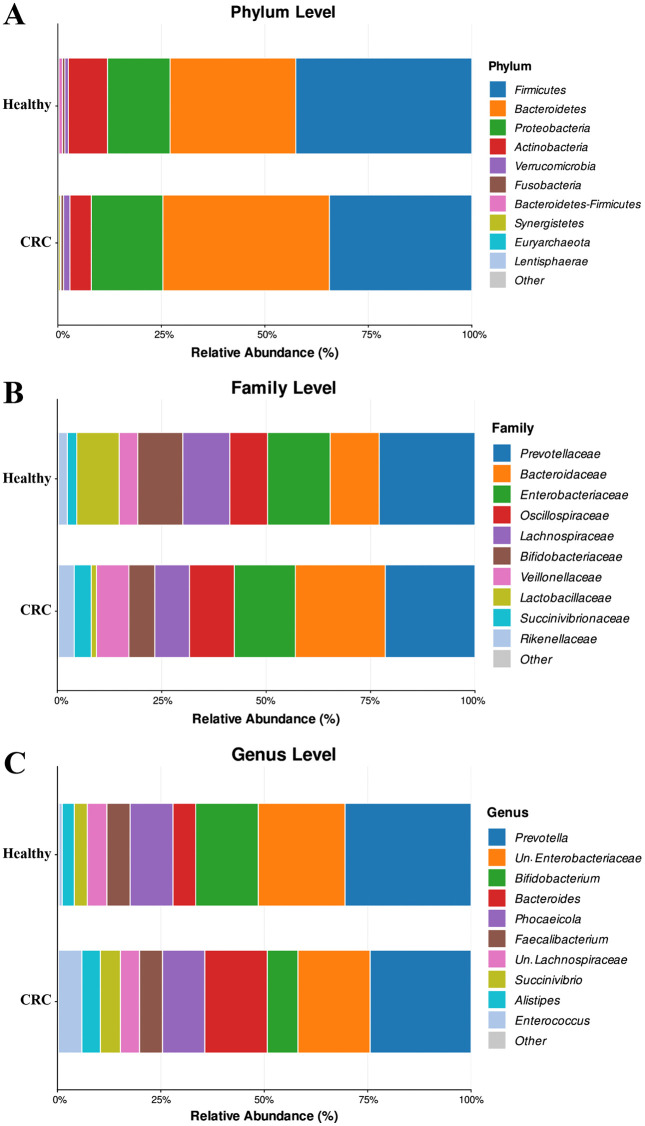
The stacked bar plots illustrate the relative composition of taxa in the CRC and HC groups at (A) phylum, (B) family, and (C) genus levels. The proportion of top 10 phylum, family and genera are displayed.

Taxonomic inference of the gut microbiome identified 1,276 distinct ASVs, with samples containing an average of 17,568 reads per sample. These ASVs were distributed across 15 phyla, 26 classes, 40 orders, 97 families, and 236 genera, reflecting broad microbial diversity. Across all samples, the microbial community was predominantly composed of Firmicutes (39.88%), Bacteroidetes (33.55%), Proteobacteria (15.8%), and Actinobacteria (8.06%), with smaller contributions from Verrucomicrobia (1.05%) and Fusobacteria (0.61%). Compared to HC, CRC patients showed an higher relative abundance of Bacteroidetes (CRC: 40.22% vs. HC: 30.36%), Proteobacteria (CRC: 17.26% vs. HC: 15.11%), and Verrucomicrobia (CRC: 1.5% vs. HC: 0.84%) along with a decreased abundance of Firmicutes (CRC: 34.38% vs. HC: 42.52%) and Actinobacteria (CRC: 5.2% vs. HC: 9.44%) (**Fig 3A**) (**S1 Table**).

At the family level, Prevotellaceae (17.67%), Bacteroidaceae (11.74%), Enterobacteriaceae (11.69%), and Lachnospiraceae (8.10%) were identified as the most prevalent families (**S2 Table**). In the CRC group, elevated relative abundances were observed in Bacteroidaceae, Veillonellaceae, and Succinivibrionaceae, whereas Bifidobacteriaceae, Lactobacillaceae, Streptococcaceae, and Lachnospiraceae, exhibited a reduction in relative abundance (**Fig 3B**). Consistently, genus-level analysis revealed increased abundances of *Bacteroides*, *Phocaeicola, Succinivibrio, and Alistipes* (phylum Bacteroidetes and Proteobacteria) in CRC, alongside reduced levels of *Bifidobacterium* and *Streptococcus* (phylum Actinobacteria and Firmicutes) (**Fig 3C**) (**S3 Table**).

### 3.4. Identification of differentially abundant bacterial taxa

**Fig 4:**
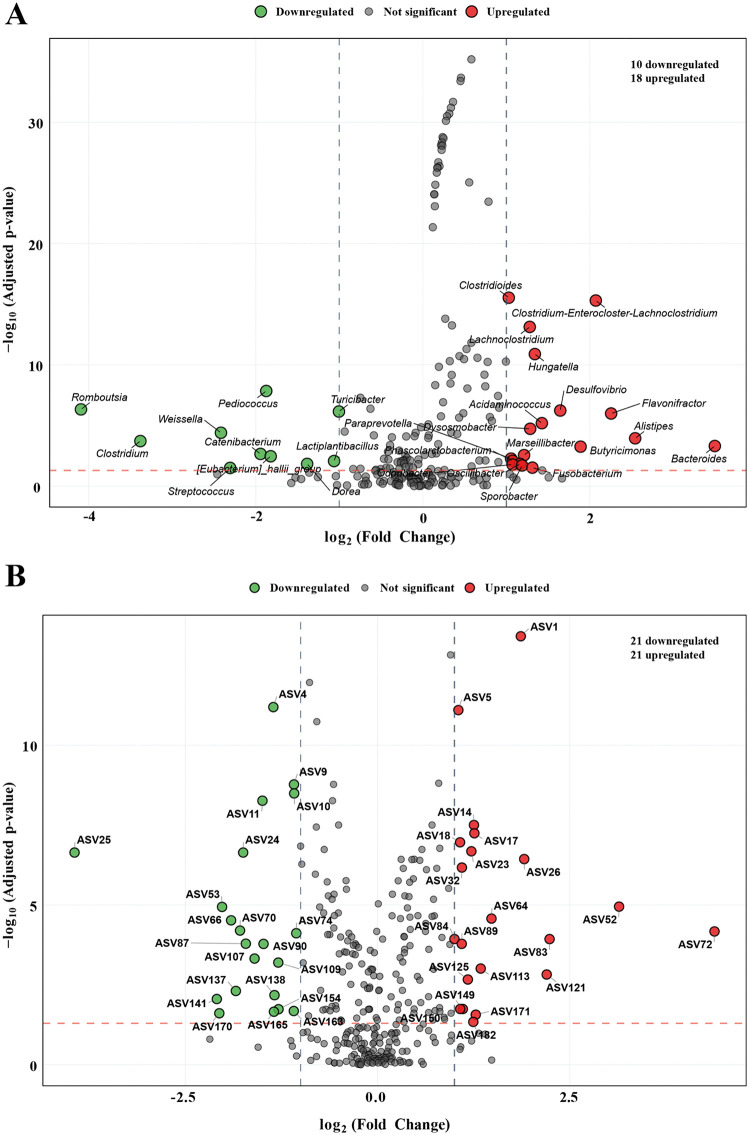
Differentially abundant taxa (A) genus and (B) species detected by ZIGMM (|log_2_FC| > 1.0 and adj.p < 0.05) between CRC and HC individuals.

To identify significantly differentially abundant bacterial taxa, ZIGMM was applied using thresholds of adjusted *p*-value < 0.05 and |log_2_FC| > 1. Phylum level analysis revealed significant enrichment of Actinobacteria (log_2_FC = −1.744, adj.*p* = 0.0074) in HC group, while Fusobacteria (log_2_FC = 1.13, adj.*p* = 0.0411) was significantly enriched in CRC group (**S1 Fig**). At the family level, 15 taxa showed clear significant group-specific differences including Peptostreptococcaceae, Lactobacillaceae, Streptococcaceae, and Clostridiaceae which were enriched in HC group, whereas Bacteroidaceae, Rikenellaceae, Acidaminococcaceae, and Odoribacteraceae were more abundant in CRC group (**S2 Fig**), indicating a marked shift in gut microbial composition associated with CRC (**S4 Table**). At the genus level, 28 genera emerged as differentially abundant with 18 enriched and 10 depleted (**Fig 4A**) in CRC including *Romboutsia* (Peptostreptococcaceae; log_2_FC = −4.09, adj.*p* = 4.68E-07), *Bacteroides* (Bacteroidaceae; log_2_FC = 3.49, adj.*p* = 0.00048), *Clostridium* (Clostridiaceae; log_2_FC = −3.38, adj.*p* = 6.66E-05), *Alistipes* (Rikenellaceae; log_2_FC = 2.54, adj.*p* = 0.00011), *Streptococcus* (Streptococcaceae; log_2_FC = −2.30, adj.*p* = 0.0309), *Flavonifractor* (Firmicutes; log_2_FC = 2.25, adj.*p* = 1.02E-06). Complete genus-level results are compiled in **S5 Table**. For identifying differentially abundant bacterial species, ZIGMM is used at the ASV level, revealing 42 differentially abundant ASVs between CRC and HC groups (**Fig 4B**), which were subsequently mapped to corresponding bacterial species.

Among these 42 species, 21 were enriched in CRC patients, while the remaining 21 were depleted compared to HCs including *Bacteroides fragilis (Bacteroides;* log_2_FC=2.23, adj.*p* = 0.00011)*, Bacteroides uniformis (Bacteroides;* log_2_FC = 2.20, adj.*p* = 0.0014)*, Bacteroides ovatus (Bacteroides;* log_2_FC=3.14, adj.*p* = 1.12E-05)*, Flavonifractor plautii (Flavonifractor;* log_2_FC=1.90, adj.*p* = 3.65E-07) and *Clostridium celatum (Firmicutes;* log_2_FC = −2.02, adj.*p* = 1.14E-05). All 42 differential bacterial species information are compiled in **S6 Table**. The top 10 bacterial taxa were initially considered among the 42 significantly differentially abundant species. Among these top 10, taxa having greater effect size, species-level taxonomic annotations, and support from published articles about their associations with CRC, were prioritized for further investigation. Consequently, four bacterial taxa (*Bacteroides fragilis, Bacteroides ovatus, Bacteroides uniformis,* and *Flavonifractor plautii*) were selected. Although *Bacteroides uniformis* and *Bacteroides ovatus* are regarded as commensals, their association with CRC has been reported in several previous studies [19,84–103], and their inclusion is further supported by evidence that commensal bacteria may exhibit opportunistic pathogenic behavior depending on host immune status and environmental conditions, and may enhance the virulence of co-occurring pathogens in mixed infections [104,105]. The top 10 most differentially abundant bacterial taxa, ranked by adjusted p-value and log_2_FC, are presented in **Table 1**.

**Table 1:** Top 10 significantly altered gut microbiota between CRC and HCs, based on log_2_FC and adjusted *p*-value.

*Phylum*	*Family*	*Genus*	*Species*	*log2FC*	*Adjusted* *P-value*
Bacteroidetes	Bacteroidaceae	*Bacteroides*	*spp*.	4.38	6.70E-05
Firmicutes	Peptostreptococcaceae	*Romboutsia*	*unclassified*	−3.94	2.28E-07
Bacteroidetes	Bacteroidaceae	*Bacteroides*	*ovatus*	3.14	1.12E-05
Bacteroidetes	Bacteroidaceae	*Bacteroides*	*fragilis*	2.23	0.0001
Bacteroidetes	Bacteroidaceae	*Bacteroides*	*uniformis*	2.20	0.001
Actinobacteria	Bifidobacteriaceae	*Bifidobacterium*	*unclassified*	−2.09	0.0087
Firmicutes	Lachnospiraceae	*Blautia*	*unclassified*	−2.05	0.024
Firmicutes	Clostridiaceae	*Clostridium*	*celatum*	−2.02	1.14E-05
Firmicutes	Oscillospiraceae	*Flavonifractor*	*plautii*	1.90	3.65E-07
Firmicutes	Clostridiaceae	*Clostridium*	*unclassified*	−1.90	3.01E-05

### 3.5. Subtractive genomic approach for bKGs identification in CRC-associated bacteria

Subtractive genomic analysis was conducted on the four CRC-associated bacterial species identified through differential abundance analysis-*Bacteroides fragilis*, *Bacteroides uniformis*, *Bacteroides ovatus*, and *Flavonifractor plautii*. The complete reference proteomes for these organisms were retrieved from the UniProt database using accession IDs UP000006731, UP000004110, UP000473905, and UP000029585, respectively. These reference proteomes served as functional approximations as the actual bacterial strains present in the CRC samples may differ from the reference strains. A summary of the proteomic characteristics for each species is provided in **Table 2**.

**Table 2:** Steps involved in selecting key proteins of identified CRC-associated bacteria through subtractive genomics analysis.

S.N.	Subtractive approaches	*BaF* (strain: ATCC 25285)	*BaU* (strain: ATCC 8492)	*BaO* (strain: BIOML-A134)	*FlaP* (strain: 1_3_50AFAA)
1.	Proteome (Total sequence)	4234	4618	6058	4332
2.	Non-paralogous sequences	4085	4419	5607	4047
3.	Non-homologous proteins	3737	4084	5240	3614
4.	Essential proteins	665	607	658	322
5.	Essential metabolic proteins	152	153	155	115
6.	Subcellular localization (Cytoplasmic)	111	118	119	90
7.	PPI networking analysis	5	5	5	5
8.	Drug target analysis	5	5	5	5

*BaF: Bacteroides fragilis, BaU: Bacteroides uniformis, BaO: Bacteroides ovatus, FlaP: Flavonifractor plautii.

After collecting the bacterial proteomes, paralogous entries were removed using the CD-HIT with a 60% identity cutoff, identifying 149 paralogs in *BaF*, 199 in *BaU*, 451 in *BaO*, and 285 in *FlaP*. The remaining non-paralogous proteins were screened against the human proteome using BLASTp, applying a sequence identity threshold of ≤30%, an E-value cutoff of ≤1 × 10 ⁻ ^5^, and a minimum query coverage of ≥70%, yielding 3,737 non-homologous proteins in *BaF*, 4,084 in *BaU*, 5,240 in *BaO*, and 4,047 in *FlaP*. These non-homologous proteins were then considered as candidates for downstream drug-target prioritization.

To determine the essentiality, the non-homologous protein sets were screened against the DEG10 database, applying thresholds of sequence identity ≥40%, query coverage ≥80%, bit score ≥100, and E-value ≤1 × 10 ⁻ ^10^, identifying 665 essential proteins in *BaF*, 607 in *BaU*, 658 in *BaO*, and 322 in *FlaP*. Metabolic pathway analysis of these essential non-homologous proteins was performed using the eggNOG-mapper server. Pathway analysis results showed the number of pathways that were shared with human pathways, and the number of proteins that are unassigned to KO identifiers. For *BaF*, out of 665 essential non-homologous proteins 230 proteins lacked KO assignments, 129 KO-annotated proteins were not linked to any pathway, and 166 pathways overlapped with those in humans. In *BaU*, 183 proteins lacked KO identifiers, 129 KO-assigned proteins did not map to any pathways, and 166 pathways were shared with human pathways. For *BaO*, 213 proteins lacked KO annotation, 137 KO-assigned proteins had no associated pathways, and 152 pathways were common to human pathways. In the case of *FlaP*, 32 proteins lacked KO identifiers, 87 KO-assigned proteins showed no pathway mapping, and 140 pathways were shared with human pathways. After eliminating the shared pathways, the analysis identified 72 unique bacterial pathways in *BaF*, 66 in *BaU*, 62 in *BaO*, and 68 in *FlaP*, and their corresponding unique bacterial pathway proteins are 152 in *BaF*, 153 in *BaU*, 155 in *BaO*, and 115 in *FlaP*. These pathway proteins were subsequently subjected to subcellular localization analysis (S7–S10 Tables). Prediction of subcellular localization offered valuable insights into the functional characteristics and therapeutic potential of these proteins.

Subcellular localization analysis of the pathway proteins revealed that in *BaF*, 111 were cytoplasmic, 21 cytoplasmic membrane-associated, 2 periplasmic, and 18 proteins with unknown localization. In *BaU*, 118 were cytoplasmic, 20 cytoplasmic membrane-associated, 1 periplasmic, 1 extracellular, and 13 proteins of unknown localization. For *BaO*, 119 were cytoplasmic, 22 cytoplasmic membrane-associated, 3 periplasmic, and 11 of unknown localization. Finally, *FlaP* exhibited 90 cytoplasmic proteins, 18 cytoplasmic membrane-associated, and 7 proteins of unknown localization (**S11 Table**).

**Fig 5:**
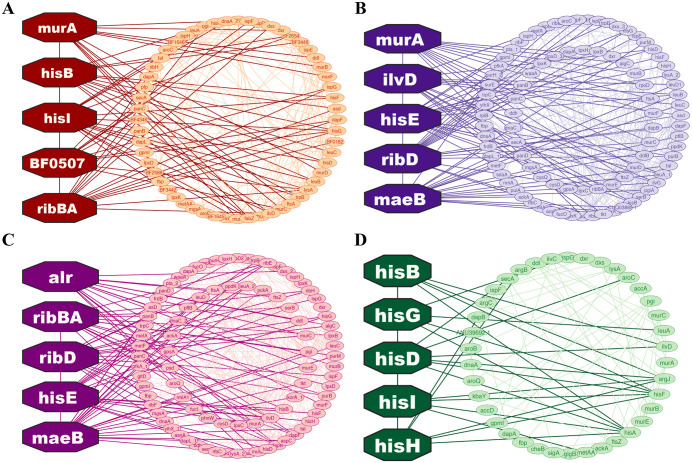
Protein-protein interaction networks depicting the top five hub proteins for (A) Bacteroides fragilis, (B) Bacteroides uniformis, (C) Bacteroides ovatus, and (D) Flavonifractor plautii.

As cytoplasmic proteins are frequently used as therapeutic targets [106], only proteins located in the cytoplasm were selected for subsequent analyses. Protein-protein interaction networks were generated for these cytoplasmic proteins in each bacterium using the STRING platform with medium interaction confidence (0.4). The resulting networks were visualized in Cytoscape, and the top five hub proteins for each bacterium were identified based on Degree topological measure using the cytoHubba plugin in Cytoscape (Fig 5). The top 5 hub proteins from each of the four bacteria yielded 20 proteins in total, from which duplicate entries appearing across multiple species were removed, resulting in a set of unique proteins. After removing overlapping proteins and proteins with unavailable structure information, a final set of 10 proteins (*ribD, ribBA, murA, alr, hisI, hisE, hisD, hisG, hisH,* and *hisB)* were retained as bKGs for subsequent analyses (S12 Table). These bKGs are conserved essential bacterial genes and are identified here as putative antibacterial targets, since they are essential for survival, have low similarity to human homologs [107,108].

### 3.6. Druggability analysis of bKGs

The druggability of each target protein was evaluated by identifying potential ligand-binding pockets and ranking them according to their predicted suitability for small-molecule binding. Multiple binding pockets were detected for all selected proteins. For each pocket, structural and physicochemical properties, including pocket volume (Å³), surface area (Å²), drug score, and simple score, were calculated. Pockets with a drug score ≥ 0.5 were considered to have a greater likelihood of being modulated by drug-like molecules. All ten bKGs had at least one pocket with drug scores ≥ 0.5, confirming their predicted druggability and suitability as targets for small-molecule binding. The druggability parameters for the selected pockets are (see S3–S12 Figs for pocket visualization) compiled in **Table 3**.

**Table 3:** Druggability information of bKGs (No. of binding pocket, highest binding pocket, pocket volume, surface area, drug score and simple scores).

Gene	Uniprot_id	No. of binding pockets	Highest binding pocket	Volume (Å^3^)	Surface (Å^2^)	Drug score	Simple score
*ribD*	A0A5M5DBX0	7	P_0	1232.19	1694.05	0.81	0.62
*ribBA*	Q5LHT1	11	P_0	2460.77	3128.46	0.8	0.63
*murA*	A0ABC9NCX7	12	P_0	1458.94	1408.86	0.81	0.61
*alr*	A0A5M5DT22	35	P_0	2701.34	2771.96	0.81	0.62
*hisI*	Q5LBD8	5	P_1	378.82	739.81	0.74	0.21
*hisE*	A0ABC9N566	6	P_0	670.08	1029.33	0.81	0.49
*hisD*	A0A096CN98	10	P_0	1978.71	2316.44	0.81	0.63
*hisG*	A0A096BB53	10	P_0	719.23	930.74	0.81	0.52
*hisH*	A0A096BA31	6	P_0	253.63	474.70	0.65	0.13
*hisB*	A0A096DFI2	7	P_1	203.97	305.98	0.54	0

### 3.7. Molecular docking

**Fig 6:**
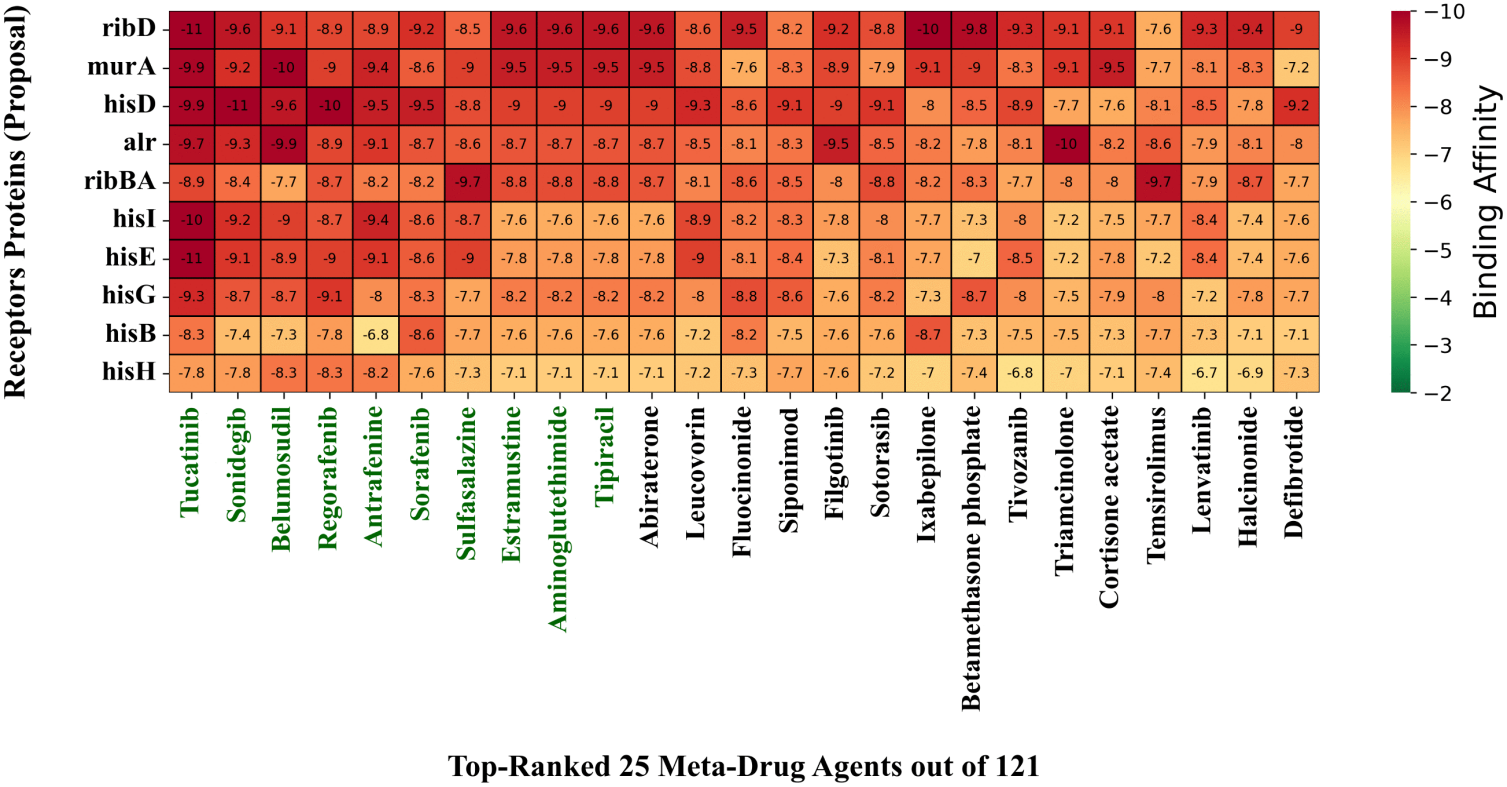
Heatmap representation of binding affinity scores between bacterial key gene (Y-axis) and candidate therapeutic compounds (X-axis, top 25 ranked). The matrix illustrates interaction strengths for 10 prioritized protein targets against 25 screened molecules. Green shading identifies the 10 compounds with superior binding affinity scores.

Molecular docking analyses were performed to assess the interaction strength between the prioritized target proteins and the curated ligand set. 3D-structures of the 10 selected bKGs (*ribD, ribBA, murA, alr, hisI, hisE, hisD, hisG, hisH,* and *hisB)* were obtained from the AlphaFold Protein Structure Database and SWISS-MODEL, and these structural models were subsequently used for molecular docking. A total of 121 candidate compounds associated with CRC were compiled from the DrugBank (**S13 Table**). Both protein and ligand structures were prepared according to the procedure outlined in the Methods section. Molecular docking was then executed for all protein-ligand combinations, generating BASs (kcal/mol). The 25 highest-scoring interactions were visualized in a matrix heatmap. Based on these results, the top 10 ligands Tucatinib, Sonidegib, Belumosudil, Regorafenib, Antrafenine, Sorafenib, Sulfasalazine, Estramustine, Aminoglutethimide, and Tipiracil-highlighted in green in **Fig 6**, were selected as the most promising preliminary drug candidates for in-depth investigation against the proposed bKGs. The identified candidates represent computational predictions derived from a multi-step inference pipeline; experimental validation is therefore essential before any therapeutic relevance can be established.

### 3.8. Drug likeness and ADME/T analysis

Drug-likeness of the 10 lead drug candidates showing the strongest predicted average binding affinity toward the selected targets was evaluated using Lipinski’s Rule of Five (ROF). This guideline considers molecular properties such as molecular weight (<500 Da), lipophilicity (logP ≤ 5), and limits on hydrogen-bond donors (≤5) and acceptors (≤10). Among 10 lead candidates, Belumosudil, Sulfasalazine, Aminoglutethimide, and Tipiracil are the promising preliminary candidates that follow all the drug-like characteristics without permitting any single violation. A comprehensive summary of the drug likeness properties for each compound is provided in **Table 4**.

**Table 4:** Drug likeness properties of top 10 drug candidates with highest binding affinities.

Compounds	Molecular Weight (<500)	LogP (≤ 5)	H-bond Donors (≤ 5)	H-bond Acceptors (≤ 10)	Polar Surface area (Å^2^)	Lipinski’s Rule	
						Follow	Violation
TUCATINIB	480.532	5.094	2	10	110.85	3	1
SONIDEGIB	485.506	5.822	1	6	63.69	3	1
BELUMOSUDIL	452.518	4.82	3	8	104.82	4	0
REGORAFENIB	482.821	5.689	3	7	92.35	3	1
ANTRAFENINE	588.552	6.995	1	6	57.7	3	1
SORAFENIB	464.831	5.55	3	7	92.35	3	1
SULFASALAZINE	398.4	3.702	3	9	141.31	4	0
ESTRAMUSTINE	440.411	5.182	1	4	49.77	3	1
AMINOGLUTETHIMIDE	232.283	1.353	3	4	72.19	4	0
TIPIRACIL	242.666	0.29	3	6	92.81	4	0

Evaluation of the ADME and toxicity profiles highlighted Sulfasalazine, Aminoglutethimide, and Tipiracil as the most promising preliminary therapeutic candidates based on computational predictions. All three compounds exhibited high human intestinal absorption (>90%), indicating strong predicted oral bioavailability and efficient absorption. Their Blood-Brain Barrier permeability was limited (BBB < 0.3) and not penetrable into the CNS, which is desirable for minimizing potential neurotoxic effects. Toxicity assessment further supported the overall predicted safety profiles of these compounds. Overall, the combination of computationally predicted absorption characteristics, constrained BBB entry, and acceptable toxicity profiles underscores their potential as preliminary drug candidates, requiring experimental validation for establishing therapeutic relevance. A comprehensive summary of the pharmacokinetic and toxicity profiles for each compound is provided in **S14 Table**.

### 3.9. Validation of docking protocol using decoy molecules

To validate the molecular docking protocol, the BASs of the selected drug compounds were compared against the average BASs of corresponding decoy molecules for each target protein (S15 Table). In all cases, the selected compounds demonstrated superior binding affinity scores compared to the decoy molecules (**S16 Table**), confirming that the observed protein-ligand interactions were not random. These results support the reliability of the docking protocol and confirm that the selected compounds were distinguishable from inactive decoy molecules based on their binding affinity scores, validating the specificity of the docking results.

### 3.10. Binding site assessment of final selected complexes

The docking poses of the final selected complexes were cross-referenced with the predicted pocket residues of the proteins. For *ribBA*, the docked ligand occupied the top predicted pocket, with all interacting residues confirmed as members of the predicted pocket. For *ribD*, DoGSiteScorer predicted 3 pockets with comparable drug scores (0.81, 0.8, and 0.82), indicating multiple potential binding pockets across the surface. The docked ligand occupied second ranked pocket with all interacting residues fully contained within the predicted cavity (S17 Table). This reflects blind docking independently binding within the computationally predicted druggable pockets of the target proteins, providing a computational justification of the identified docking sites.

### 3.11. Molecular dynamics simulation

**Fig 7:**
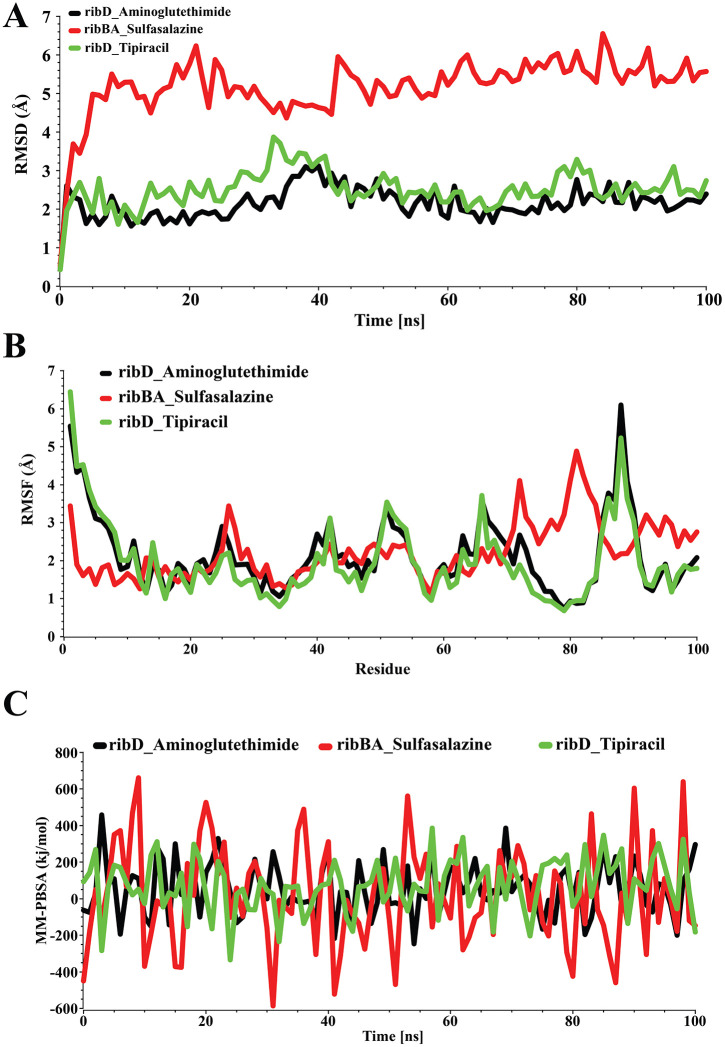
MD simulation results: (A) the root means square deviation (RMSD), (B) the root mean square fluctuation (RMSF), and (C) the binding free energy (MM-PBSA) of the top-ranked drug-target complexes with 3 candidate drugs for a duration of 100-ns simulation.

MD simulation analysis of the three selected protein-ligand complexes (*ribBA*_sulfasalazine, *ribD*_aminoglutethimide, and *ribD*_tipiracil) with superior docking scores, satisfaction of Lipinski Rule of Five criteria, and favorable ADME/T profiles, was performed to assess conformational stability and binding consistency of each compound throughout the 100 ns trajectory. The evaluation focused on key metrics including RMSD, RMSF, and MM-PBSA binding free energy. RMSD indicates the average atomic displacement over time, reflecting complex stability; as depicted in **Fig 7**. With average RMSD values of 2.145 Å, and 2.518 Å *ribD*_aminoglutethimide and *ribD*_tipiracil exhibited better stability than *ribBA*_sulfasalazine (average RMSD 5.189 Å). Here, *ribD*_aminoglutethimide exhibited the least fluctuation, suggesting greater stability (Fig 7A). RMSF analysis revealed that *ribD*-Tipiracil exhibited the lowest residue flexibility with an average value of 1.980 Å, followed by *ribD*-Aminoglutethimide (2.140 Å) and *ribBA*-Sulfasalazine (2.234 Å), indicating more consistent and stable interactions (Fig 7B). MM-PBSA binding free energy, revealed good stability with average scores of 56.24 kJ/mol (*ribD*_aminoglutethimide), 18.67 kJ/mol (*ribBA*_salfasalazine), and 74.21 kJ/mol (*ribD*_tipiracil). *ribD*_aminoglutethimide demonstrated good binding, followed by *ribD*_tipiracil, with *ribBA*_salfasalazine showing comparatively weaker interaction (Fig 7C).

## 4. Discussion

The gut microbiome has been increasingly associated with colorectal carcinogenesis, with studies suggesting its potential involvement in inflammatory processes and cellular signaling pathways [109]. Throughout cancer development, a complex interplay has been observed among the gut microbial community, the tumor associated microbiota, and the host immune system [110,111]. CRC pathogenesis represents a multifactorial process associated with inherited genetic alterations, lifestyle-related risk factors, and compositional shifts in gut microbiota. Accumulating evidence suggests that gut microbial alterations may be associated with treatment resistance and tumor evolution, emphasizing the growing need to develop microbiome-guided strategies for cancer management [20,112]. Therapeutic agents that act on human proteins and those that target microbial proteins provide two complementary avenues of treatment. Modulating host proteins may influence internal biological pathways, whereas targeting bacterial proteins may help in reshaping gut microbial activity, which may affect metabolism and disease outcomes [113–115]. This study utilized gut microbiome derived 16S rRNA sequencing data to identify bKGs as putative antibacterial targets within CRC-associated bacterial taxa and to screen candidate drug compounds through computational drug repurposing. Our analysis revealed significant differences in the overall microbial profile between CRC patients and the HC group. The observed phylum-level alterations in CRC specifically, increased Fusobacteria (previously associated with colorectal carcinogenesis) abundance contrasted with reduced Actinobacteria (known to produce SCFA and possesses anti-inflammatory properties) represent compositional shifts reported in CRC-associated microbiome studies [116]. At the family level, high enrichment of the families Bacteroidaceae, Rikenellaceae, and Acidaminococcaceae in CRC [91,117,118] and high enrichment of Bifidobacteriaceae, Lactobacillaceae, and Lachnospiraceae [119–121] in HC group corroborates findings with other studies. Differential abundance analysis uncovered 42 bacterial species that were significantly altered in CRC compared to HC individuals. Bacterial species including *Bacteroides fragilis, Flavonifractor plautii, Bacteroides uniformis,* and *Bacteroides ovatus* were significantly enriched in CRC group, while taxa including *Romboutsia, Bifidobacterium,* and *Blautia* remained significantly depleted. The depletion of *Romboutsia, Bifidobacterium,* and *Blautia* consistent with emerging literature on gut microbiota compositional alterations associated with CRC [119,122,123]. The elevated abundance of *Bacteroides* species observed in CRC patients compared to HC subjects is well documented in several microbiome studies [124,125]. Enterotoxigenic *Bacteroides fragilis* has emerged as a potential diagnostic biomarker for CRC and has been associated with unfavorable prognosis result [126]. *Bacteroides fragilis* has been reported to promote tumor development through various mechanisms, including modulation of NF-κB signaling, induction of DNA damage, enhancement of polyamine metabolism, stimulation of TH17 immune responses, and activation of stem cell functions [127]. *Flavonifractor plautii*, a major flavonoid-degrading bacterium, is known for its elevated flavonoid degradation, potentially reducing the beneficial effects and bioavailability of flavonoids in CRC. Moreover, its association with the catechol cleavage pathway which processes catechols produced during flavonoid breakdown further highlights its role in gut flavonoid metabolism [88]. *Bacteroides uniformis* and *Bacteroides ovatus* were enriched in CRC in some studies [84,101] and have been associated with bacteremia which has been linked to increased CRC risk [85], though they have a report of being present as gut commensal or as member of healthy gut [128,129]. Among the 42 altered bacteria, four bacteria were selected (*Bacteroides fragilis, Bacteroides ovatus, Bacteroides uniformis,* and *Flavonifractor plautii*) for further investigation. Although *Bacteroides uniformis* and *Bacteroides ovatus* are regarded as commensals, their association with CRC has been reported in several previous studies [84–86,90–93,97,101–103], and their selection is further supported by evidence that commensal bacteria may exhibit opportunistic pathogenic behavior depending on host immune status and environmental conditions, and may enhance the virulence of co-occurring pathogens in mixed infections [104,105]. Analysis of the top four CRC-associated bacteria identified 10 conserved essential bKGs (*ribD, ribBA, murA, alr, hisI, hisE, hisD, hisG, hisH,* and *hisB*) within these taxa. These bKGs are fundamental for bacterial survival, mediating the biosynthesis of riboflavin, histidine, and cell wall component, and may serve as putative antibacterial targets, given their essentiality, low similarity to human homologs [130]. Bacterial essential genes involved in peptidoglycan biosynthesis and riboflavin metabolism have been reported to indirectly influence host immune signaling through the release of bacterial cell wall fragments and metabolic intermediates that interact with host [131,132]. Essential genes involved in histidine biosynthesis have also been reported to influence host-microbiome crosstalk [133,134], which may indicate their indirect role in disease development. The genes *ribD* and *ribBA* play critical roles in bacterial riboflavin biosynthesis, providing the precursor required for FMN and FAD cofactors that support redox processes and core metabolic functions. Their inhibition may offer a potential strategy to compromise the metabolic fitness and growth of CRC-enriched bacterial taxa [135–137]. The *his* genes collectively encode enzymes responsible for histidine biosynthesis, a highly regulated metabolic pathway in bacterial systems, disruption of these genes has been associated with histidine auxotrophy and loss of bacterial virulence supporting their candidacy as putative antibacterial targets within the CRC-associated bacterial taxa identified in this study [138,139]. The genes *murA* and *alr* encode key enzymes involved in peptidoglycan biosynthesis, rendering them attractive targets for antibacterial intervention [140–142]. Collectively, these 10 genes represent conserved essential metabolic targets without human homologs which may have potential influence on host-microbes interaction, making them potentially suitable candidates for selective antibacterial intervention against CRC-associated bacterial taxa. To identify therapeutic compounds capable of targeting the prioritized bKGs, a comprehensive in-silico drug screening analysis prioritized three compounds (Sulfasalazine, Aminoglutethimide, and Tipiracil) as antibacterial agents based on their strong predicted binding affinity against the identified bKGs, compliance with Lipinski Rule of Five drug-likeness criteria and favorable pharmacokinetic (limited BBB penetration, limited CNS penetration, high intestinal absorption) and toxicity profiles (non-mutagenic) **(see** **S14 Table**). MD simulation analysis further supported the structural stability of the selected complexes. Sulfasalazine is an anti-bacterial agent that acts on colonic bacterial azoreductase enzyme and is metabolized to the anti-inflammatory metabolite and anti-bacterial metabolites sulfapyridine [143]. Sulfasalazine is also a well-established anti-inflammatory agent, exerts anti-tumor effects by promoting apoptosis and tumor shrinkage through blockade of the plasma membrane-associated system xc^-^ cystine transporter [144]. Sulfasalazine demonstrated strong predicted binding affinity with *murA, ribBA*, and *hisE* may be suggesting potential interference with peptidoglycan and riboflavin biosynthesis in CRC-associated bacterial taxa. Aminoglutethimide and Tipiracil demonstrated favorable predicted binding affinity with *ribD*, *murA*, and *hisD*, suggesting potential influence on riboflavin and peptidoglycan biosynthesis pathways and bacterial growth. Notably, Tipiracil along with Trifluridine has been approved for use in metastatic CRC patients that have failed or are unsuitable for standard chemotherapeutic and biologic treatments as well as for patients with unresectable advanced or recurrent CRC [145], further supporting the translational relevance of these computationally identified candidates. Collectively, these multi-layered analyses provide preliminary support for exploring microbiome-guided therapeutic strategies relevant to CRC and identifying a set of putative microbial gene-drug interactions as candidates for further investigation. These findings represent computational predictions that warrant experimental validation to confirm their antibacterial efficacy, functional relevance, and therapeutic potential in the context of CRC-associated gut microbiota.

## 5. Strengths and limitations

One of the advantages of this study is the use of a comprehensive computational pipeline that integrates 16S rRNA-based microbial profiling, statistical analysis, subtractive genomics, protein-protein interaction network analysis, molecular docking and ADME/T analysis to explore bKGs as putative antibacterial targets in CRC-associated bacterial taxa and prioritize candidate therapeutics. Together, these approaches offer a systematic and integrative framework for identifying potential therapeutic targets. Despite these strengths, several limitations should be acknowledged. All conclusions are drawn from in-silico analyses and therefore require confirmation through laboratory experiments including in vitro and in vivo validation. This study focused exclusively on the bacterial targets and did not incorporate host molecular pathways. The workflow involves multiple sequential computational inferences which may introduce cumulative uncertainty. Taxonomic assignments from V4 16S amplicon sequencing are probabilistic rather than definitive, and the reference proteomes used represent functional approximations rather than actual patient-derived strains. The drug compounds were sourced from DrugBank based on CRC association for repurposing investigation and may not represent the most appropriate library for bacterial enzyme inhibition. Molecular docking results reflect predicted binding affinities based on computational models, and evidence of drug efficacy needs experimental confirmation. Additionally, blind docking approach was employed with no benchmarking against known inhibitors due to the absence of experimentally resolved structures of the target proteins, and docking was performed using a single engine (AutoDock Vina) without replication across alternative platforms. Furthermore, gut-targeted antibacterial parameters including intestinal luminal stability, bacterial membrane uptake, and activity under anaerobic conditions could not be included in the ADME/T analysis, due to computational limitations. Finally, the dataset comprises a relatively modest single cohort without available clinical metadata, and the broader biological influence of the identified bKGs on host metabolic processes remains to be established through functional and longitudinal studies.

## 6. Conclusions

This study employed an integrative in silico framework comprising 16S rRNA-based microbial profiling, subtractive genomics, PPI network analysis, molecular docking and ADME/T analysis to investigate gut microbiota compositional alteration associated with CRC and to identify bKGs in CRC-associated gut microbiota as potential targets for antibacterial therapy. Analysis of 16S rRNA sequencing data revealed significant alterations in bacterial community composition between CRC and HC groups. Differential abundance analysis identified several CRC-associated bacterial taxa, including *Bacteroides fragilis, Bacteroides uniformis, Bacteroides ovatus*, and *Flavonifractor plautii* which have been previously associated with CRC in observational studies. Using a subtractive genomics approach and PPI network analysis, ten essential bKGs (*ribD, ribBA, murA, alr, hisI, hisE, hisD, hisG, hisH* and *hisB*) were identified from these CRC-associated bacterial taxa as putative antibacterial targets as these proteins are of conserved essential bacterial metabolic proteins. Subsequent computational drug screening identified ten candidate compounds, of which Sulfasalazine, Aminoglutethimide, and Tipiracil exhibited favorable in silico pharmacokinetic and toxicity profiles that support their potential suitability for repurposing as antibacterial agents in CRC. Finally, this study proposes a microbiome-guided strategy that integrates bKGs identification with computational drug repurposing to explore antibacterial therapeutic options relevant to CRC-associated microbiome. Targeting CRC-associated altered bacteria and their essential bKGs may offer a promising complementary treatment avenue. However, experimental validation and clinical studies are necessary to confirm the efficacy, safety and translational potential of the identified drug candidates while future functional and patient-specific microbiome analyses may further support personalized CRC interventions.

## Supporting information

S1 Fig Phylum level differential analysis between HC and CRC group. (TIF)

S2 Fig Differentially Abundant Family (|log2FC| > 1 and adj.p-value<0.05). (TIF)

S3 Fig *ribD* protein (pocket with highest drug score). (TIF)

S4 Fig *ribBA* protein (pocket with highest drug score). (TIF)

S5 Fig *murA* protein (pocket with highest drug score). (TIF)

S6 Fig *alr* protein (pocket with highest drug score). (TIF)

S7 Fig *hisI* protein (pocket with highest drug score). (TIF)

S8 Fig *hisE* protein (pocket with highest drug score). (TIF)

S9 Fig *hisH* protein (pocket with highest drug score). (TIF)

S10 Fig *hisG* protein (pocket with highest drug score). (TIF)

S11 Fig *hisD* protein (pocket with highest drug score). (TIF)

S12 Fig *hisB* protein (pocket with highest drug score). (TIF)

S1 Table Percentage of Each phylum. (XLSX)

S2 Table Percentage of each family’s contribution to the overall microbiome. (XLSX)

S3 Table Percentage of each genera’s contribution to the overall microbiome. (XLSX)

S4 Table Differentially abundant microbial family (|log2FC| > 1 and adj.p-value<0.05). (XLSX)

S5 Table Differentially abundant microbial genera (|log2FC| > 1 and adj.p-value<0.05). (XLSX)

S6 Table 42 differentially abundant bacteria (|log2FC| > 1 and adj.p-value<0.05). (XLSX)

S7 Table Unique pathogenic pathway proteins of *BaF.* (XLSX)

S8 Table Unique pathogenic pathway proteins of *BaU.* (XLSX)

S9 Table Unique pathogenic pathway proteins of *BaO.* (XLSX)

S10 Table Unique pathogenic pathway proteins of *FlaP.* (XLSX)

S11 Table Proteins of 4 bacteria with different locations. (XLSX)

S12 Table Key-genes selection using degree method. (XLSX)

S13 Table Drug collection details. (XLSX)

S14 Table Pharmacokinetic and toxicity profiles of the top 10 drugs with highest binding affinity. (DOCX)

S15 Table The identifiers of the Decoy molecules generated for SULFASALZINE, AMINOGLUTETHIMIDE, and TIPIRACIL. (DOCX)

S16 Table The average binding affinity scores (BASs) of the decoy molecules and the BASs of suggested drug candidates in kcal/mol with the receptors. (DOCX)

S17 Table The list of ligand binding residues and pockets residues. (DOCX)
