# Supplementary material for: In-silico identification of bacterial key-genes directly or indirectly associated with the development and progression of colorectal cancer for exploring anti-bacterial agents
Source: PLoS One. 2026 Jun 26;21(6):e0343565. doi: 10.1371/journal.pone.0343565 (PMC13308813; doi:10.1371/journal.pone.0343565)
Supplement: S14 Table — (DOCX) [file pone.0343565.s026.docx]

| **Compounds** | **Absorption** | | | **Distribution** | | **Metabolism** | | **Excretion** | **Toxicity** | | |
| --- | --- | --- | --- | --- | --- | --- | --- | --- | --- | --- | --- |
|  | **Caco-2 (logPaap)** | **HIA** | **P-gp1 Inhibitor** | **BBB** | **CNS (logPS)** | **CYP3A4 Inhibitor** | **CYP2D6 inhibitor** | **TC (logml)** | **AMES** | **Minnow toxicity** | **Rat (Acute)** |
|  |  |  |  |  |  |  |  |  |  |  |  |
|  |  |  |  |  |  |  |  |  |  |  |  |
| TUCATINIB | -5.21 | Absorbed | Inhibitor | Non-Penetrable | -2.1 | Inhibitor | Non-Inhibitor | 5.43 | Safe | 5.89 | 2.99 |
| SONIDEGIB | -4.97 | Absorbed | Inhibitor | Penetrable | -2.03 | Non-Inhibitor | Non-Inhibitor | 2.56 | Safe | 5.81 | 2.71 |
| BELUMOSUDIL | -5.48 | Absorbed | Inhibitor | Non-Penetrable | -1.84 | Inhibitor | Inhibitor | 5.39 | Toxic | 6.87 | 2.87 |
| REGORAFENIB | -4.89 | Absorbed | Non-Inhibitor | Penetrable | -2.3 | Non-Inhibitor | Inhibitor | 8.13 | Safe | 5.07 | 2.41 |
| ANTRAFENINE | -5.12 | Absorbed | Non-Inhibitor | Penetrable | -2.3 | Non-Inhibitor | Inhibitor | 8.13 | Safe | 5.07 | 2.41 |
| SORAFENIB | -4.86 | Absorbed | Non-Inhibitor | Penetrable | -2.1 | Non-Inhibitor | Inhibitor | 7.02 | Safe | 4.96 | 2.31 |
| SULFASALAZINE | -6.2 | Absorbed | Non-Inhibitor | Non-Penetrable | -3.51 | Non-Inhibitor | Non-Inhibitor | -0.03 | Safe | 4.94 | 1.54 |
| ESTRAMUSTINE | -4.98 | Absorbed | Non-Inhibitor | Penetrable | -2.05 | Non-Inhibitor | Non-Inhibitor | 7.26 | Toxic | 3.92 | 2.84 |
| AMINOGLUTETHIMIDE | -5.12 | Absorbed | Non-Inhibitor | Non-Penetrable | -2.57 | Non-Inhibitor | Non-Inhibitor | 5.05 | Safe | 3.91 | 3.41 |
| TIPIRACIL | -6.04 | Absorbed | Non-Inhibitor | Non-Penetrable | -2.39 | Non-Inhibitor | Non-Inhibitor | 8.84 | Safe | 3.55 | 2.29 |

**S14 Table: Pharmacokinetic and toxicity profiles of the top 10 drugs with highest binding affinity.**
