# Supplementary material for: In-silico identification of bacterial key-genes directly or indirectly associated with the development and progression of colorectal cancer for exploring anti-bacterial agents
Source: PLoS One. 2026 Jun 26;21(6):e0343565. doi: 10.1371/journal.pone.0343565 (PMC13308813; doi:10.1371/journal.pone.0343565)
Supplement: S15 Table — (DOCX) [file pone.0343565.s027.docx]

## S15 Table. The identifiers of the Decoy molecules generated for SULFASALZINE, AMINOGLUTETHIMIDE, and TIPIRACIL.

| **Target Receptors** | **Generated Decoys** |
| --- | --- |
| SULFASALZINE | ZINC32011294, ZINC2265379909, ZINC13526852, ZINC1688110, ZINC90032, ZINC71373305, ZINC3768018, ZINC26378091, ZINC102947963, ZINC83260603, ZINC2383753676, ZINC83301338, ZINC39407975, ZINC141201003, ZINC34279054, ZINC2356543068, ZINC2555664, ZINC2305500017, ZINC12501100, ZINC35307761, ZINC473086980, ZINC53683979, ZINC799581450, ZINC252499606, ZINC34287769, ZINC2356646438, ZINC95617711, ZINC100498835, ZINC40646402, ZINC238050591, ZINC256006752, ZINC100829654, ZINC2010835757, ZINC5688736, ZINC306413604, ZINC17250685, ZINC79524620, ZINC36351338, ZINC40828568, ZINC4778113 |
| AMINOGLUTETHIMIDE | ZINC754348773, ZINC2559223, ZINC2053535988, ZINC2356387527, ZINC409422091, ZINC409435885, ZINC1661292, ZINC169327583, ZINC409432640, ZINC22002315, ZINC38235525, ZINC238739727, ZINC409422327, ZINC5613044, ZINC8700300, ZINC2356653145, ZINC4362000, ZINC5157859, ZINC5378768, ZINC1615166399, ZINC33821242, ZINC60220113, ZINC2265272121, ZINC44699400, ZINC263621350, ZINC238435807, ZINC409433630, ZINC15987659, ZINC238790831, ZINC198207022, ZINC33820526, ZINC33821240, ZINC118628885, ZINC821078072, ZINC78547609, ZINC4705659, ZINC5732183, ZINC1745356, ZINC2053571168, ZINC5166976 |
| TIPIRACIL | ZINC2356813368, ZINC4402138, ZINC238763304, ZINC862681865, ZINC86001857, ZINC238736329, ZINC147325578, ZINC2836023, ZINC2522619, ZINC36269635, ZINC147325794, ZINC2928182, ZINC96347212, ZINC12363565, ZINC952963706, ZINC5082467, ZINC138315226, ZINC1608878, ZINC584881655, ZINC98086462, ZINC100091209, ZINC96331665, ZINC25965839, ZINC100438806, ZINC38293778, ZINC95617704, ZINC757127972, ZINC14771814, ZINC39096116, ZINC2508225, ZINC238759630, ZINC5063405, ZINC101861116, ZINC40168045, ZINC19851863, ZINC86051650, ZINC14592178, ZINC12503707, ZINC2356606108, ZINC5157519 |
