# Supplementary material for: In-silico identification of bacterial key-genes directly or indirectly associated with the development and progression of colorectal cancer for exploring anti-bacterial agents
Source: PLoS One. 2026 Jun 26;21(6):e0343565. doi: 10.1371/journal.pone.0343565 (PMC13308813; doi:10.1371/journal.pone.0343565)
Supplement: S16 Table — (DOCX) [file pone.0343565.s028.docx]

## S16 Table. The average binding affinity scores (BASs) of the decoy molecules and the BASs of suggested drug candidates in kcal/mol with the receptors.

| **Receptors** | **Average BAS with Negative Control (Decoy)** | **BAS with Repurposed Drug Molecules** | **Drug Molecules** |
| --- | --- | --- | --- |
| *ribD* | -6.3 | -8.5 | SULFASALZINE |
|  | -6.1 | -9.6 | AMINOGLUTETHIMIDE |
|  | -5.9 | -9.6 | TIPIRACIL |
| *ribBA* | -5.9 | -9.7 | SULFASALZINE |
|  | -5.6 | -8.8 | AMINOGLUTETHIMIDE |
|  | -5.7 | -8.8 | TIPIRACIL |
| *murA* | -6.1 | -9 | SULFASALZINE |
|  | -6.2 | -9.5 | AMINOGLUTETHIMIDE |
|  | -5.8 | -9.5 | TIPIRACIL |
| *alr* | -6.2 | -8.6 | SULFASALZINE |
|  | -5.9 | -8.7 | AMINOGLUTETHIMIDE |
|  | -5.8 | -8.7 | TIPIRACIL |
| *hisI* | -5.6 | -8.7 | SULFASALZINE |
|  | -5.4 | -7.6 | AMINOGLUTETHIMIDE |
|  | -5.3 | -7.6 | TIPIRACIL |
| *hisE* | -5.5 | -9 | SULFASALZINE |
|  | -5.4 | -7.8 | AMINOGLUTETHIMIDE |
|  | -5.3 | -7.8 | TIPIRACIL |
| *hisD* | -5.8 | -8.8 | SULFASALZINE |
|  | -5.6 | -9 | AMINOGLUTETHIMIDE |
|  | -5.5 | -9 | TIPIRACIL |
| *hisG* | -5.4 | -7.7 | SULFASALZINE |
|  | -5.2 | -8.2 | AMINOGLUTETHIMIDE |
|  | -5.1 | -8.2 | TIPIRACIL |
| *hisH* | -5.8 | -7.3 | SULFASALZINE |
|  | -5.5 | -7.1 | AMINOGLUTETHIMIDE |
|  | -5.4 | -7.1 | TIPIRACIL |
| *hisB* | -5.5 | -7.7 | SULFASALZINE |
|  | -5.2 | -7.6 | AMINOGLUTETHIMIDE |
|  | -5.2 | -7.6 | TIPIRACIL |
