# Supplementary material for: In-silico identification of bacterial key-genes directly or indirectly associated with the development and progression of colorectal cancer for exploring anti-bacterial agents
Source: PLoS One. 2026 Jun 26;21(6):e0343565. doi: 10.1371/journal.pone.0343565 (PMC13308813; doi:10.1371/journal.pone.0343565)
Supplement: S17 Table — (DOCX) [file pone.0343565.s029.docx]

**S17 Table: The List of Ligand Binding Residues and Pockets Residues.**

| **Ligand Binding Residues** | **Pocket’s Residues** |
| --- | --- |
| **ribD_Aminoglutethimide** | **ribD** |
| ALA162, ASP210, GLY294, ILE169, ILE180, PRO178 | LYS_160_A, TRP_161_A, ALA_162_A, ILE_169_A, ASP_170_A, ARG_173_A, PRO_178_A, LEU_179_A, ILE_180_A, LEU_181_A, SER_182_A, VAL_190_A, ARG_194_A, ILE_200_A, VAL_202_A, GLY_203_A, ARG_204_A, ARG_205_A, THR_206_A, LEU_209_A, ASP_210_A, PRO_212_A, LEU_214_A, LEU_229_A, ASP_230_A, ARG_231_A, THR_232_A, GLU_292_A, GLY_293_A, GLY_294_A, SER_295_A, GLN_296_A, LEU_297_A, LEU_298_A, VAL_323_A |
| **ribD_Tipiracil** | **ribD** |
| ALA162, ASP210, GLY294, ILE169, ILE180, PRO178 | LYS_160_A, TRP_161_A, ALA_162_A, ILE_169_A, ASP_170_A, ARG_173_A, PRO_178_A, LEU_179_A, ILE_180_A, LEU_181_A, SER_182_A, VAL_190_A, ARG_194_A, ILE_200_A, VAL_202_A, GLY_203_A, ARG_204_A, ARG_205_A, THR_206_A, LEU_209_A, ASP_210_A, PRO_212_A, LEU_214_A, LEU_229_A, ASP_230_A, ARG_231_A, THR_232_A, GLU_292_A, GLY_293_A, GLY_294_A, SER_295_A, GLN_296_A, LEU_297_A, LEU_298_A, VAL_323_A |
| **ribBA_Sulfasalazine** | **ribBA** |
| ARG255, VAL256, ILE302, ALA340, LEU353, ARG361, GLU365 | ARG_138_A, LEU_141_A, ARG_142_A, LYS_196_A, ILE_199_A, ALA_200_A, LEU_203_A, GLU_206_A, SER_207_A, ILE_208_A, VAL_209_A, GLN_231_A, LYS_232_A, SER_233_A, VAL_254_A, ARG_255_A, VAL_256_A, HIS_257_A, SER_258_A, SER_259_A, CYS_260_A, THR_262_A, ARG_270_A, CYS_271_A, GLU_272_A, CYS_273_A, GLN_276_A, VAL_293_A, MET_295_A, GLN_297_A, GLU_298_A, GLY_299_A, ARG_300_A, ILE_302_A, LYS_307_A, ALA_310_A, TYR_311_A, GLN_314_A, GLU_315_A, TYR_318_A, ASP_319_A, THR_320_A, ALA_323_A, ASN_324_A, ARG_334_A, ASP_335_A, TYR_336_A, GLY_337_A, VAL_338_A, GLY_339_A, ALA_340_A, LEU_343_A, MET_351_A, LEU_353_A, MET_354_A, THR_355_A, ASN_356_A, ASN_357_A, PRO_358_A, LYS_360_A, ARG_361_A, ILE_362_A, GLY_363_A, LEU_364_A, GLU_365_A, ALA_366_A, TYR_367_A, LEU_369_A, GLU_370_A, ILE_371_A, GLU_373_A, ASN_374_A, ILE_377_A, GLU_378_A, ILE_379_A, LYS_380_A, PRO_381_A, TYR_384_A, ASN_385_A, GLU_386_A, ARG_387_A, TYR_388_A, LEU_389_A, LYS_390_A, THR_391_A, LYS_392_A, ASP_394_A, ARG_395_A, HIS_398_A, LEU_400_A, HIS_401_A, PHE_402_A, ASN_403_A, LYS_404_A |
